# Micronutrient Deficiencies in Patients with Decompensated Liver Cirrhosis

**DOI:** 10.3390/nu13041249

**Published:** 2021-04-10

**Authors:** Gemma Llibre-Nieto, Alba Lira, Mercedes Vergara, Cristina Solé, Meritxell Casas, Valentí Puig-Diví, Gemma Solé, Antonia Humanes, Laia Grau, Josep Maria Barradas, Mireia Miquel, Jordi Sánchez-Delgado

**Affiliations:** 1 Hepatology Unit, Digestive Disease Department, Hospital Universitari Parc Tauli, Institut d’Investigació i Innovació Parc Taulí (I3PT), 08208 Sabadell, Spain; alira@tauli.cat (A.L.); mvergara@tauli.cat (M.V.); csole@tauli.cat (C.S.); Mcasas@tauli.cat (M.C.); Vpuig@tauli.cat (V.P.-D.); mmiquel@tauli.cat (M.M.); jsanchezd@tauli.cat (J.S.-D.); 2 Departament de Medicina, Universitat Autònoma de Barcelona, 08193 Bellaterra, Spain; 3 Fundació Privada Hospital Assil de Granollers, 08402 Granollers, Spain; 4 Centro de Investigación Biomédica en Red de Enfermedades Hepáticas y Digestivas (CIBERehd), Instituto de Salud Carlos III, 28029 Madrid, Spain; 5 Gastroenterology Unit, Digestive Disease Department, Hospital Universitari Parc Tauli, Institut d’Investigacio i Innovació Parc Taulí (I3PT), 08208 Sabadell, Spain; 6 Laboratory Unit, Hospital Universitari Parc Tauli, Institut d’Investigacio i Innovació Parc Taulí (I3PT), 08208 Sabadell, Spain; gsole@tauli.cat; 7 Endocrinology and Nutrition Department, Hospital Universitari Parc Tauli, Institut d’Investigacio i Innovació Parc Taulí (I3PT), 08208 Sabadell, Spain; ahumanes@tauli.cat; 8 Statistics, Hospital Germans Trias i Pujol, Neurology Service, 08916 Badalona, Spain; lgrau.germanstrias@gencat.cat; 9 Nursing Service, Hepatology Unit, Digestive Disease Department, Hospital Universitari Parc Tauli, Institut d’Investigacio i Innovació Parc Taulí (I3PT), 08208 Sabadell, Spain; jbarradas@tauli.cat; 10 Departament de Medicina, Universitat de Vic–Universitat Central de Catalunya (UVic-UCC), 08500 Vic, Spain

**Keywords:** micronutrient deficiency, trace element deficiency, vitamin deficiency, malnutrition, decompensated cirrhosis.

## Abstract

Patients with cirrhosis often develop malnutrition and micronutrient deficiencies, leading to a worse prognosis and increased mortality. Our main goal was to assess the prevalence of micronutrient deficiencies in patients with decompensated cirrhosis. This was a prospective single-center study including 125 consecutive patients hospitalized for acute decompensation of cirrhosis (mostly of alcoholic etiology). A blood test including trace elements and vitamins was performed on admission. The main micronutrient deficiencies observed were vitamin D (in 94.5%), vitamin A (93.5%), vitamin B6 (60.8%) and zinc (85.6%). Patients in Child-Pugh class C had lower levels of vitamin A (*p* < 0.0001), vitamin E (*p* = 0.01) and zinc (*p* < 0.001), and higher levels of ferritin (*p* = 0.002) and vitamin B12 (*p* < 0.001) than those in Child-Pugh class A and B. Patients with a higher model of end-stage liver disease (MELD) score had lower levels of vitamin A (*p* < 0.0001), vitamin E (*p* < 0.001), magnesium (*p* = 0.01) and zinc (*p* = 0.001), and higher levels of ferritin (*p* = 0.002) and vitamin B12 (*p* < 0.0001). Severe hepatic insufficiency correlated with lower levels of zinc, vitamin E and vitamin A, and higher levels of vitamin B12 and ferritin.

## 1. Introduction

Malnutrition is common in patients with liver cirrhosis. Several studies have demonstrated its association with increased morbidity and mortality and decreased quality of life [1,2,3,4]. In addition, it can severely compromise liver transplant outcomes [3,5]. The prevalence of malnutrition varies depending on disease severity: it occurs in about 20% of patients with compensated disease and up to 80% of patients with severe liver failure [1,2]. 

Several authors use different definitions of the term "malnutrition", making it difficult to conduct and interpret studies properly. In general, it is defined as a decrease in skeletal muscle, visceral fat and subcutaneous tissue, but some authors also include micronutrient deficiency (vitamins and trace elements).

Vitamin and trace elements deficits are common in cirrhosis, irrespective of the etiology. Patients with liver cirrhosis have diminished vitamin reserves with respect to the general population, usually due to hepatic dysfunction, low dietary intake, low absorption and increased catabolism. In addition, malabsorption and maldigestion and use of diuretics contribute to micronutrient deficiencies [4]. Some micronutrient deficits have been studied individually in cirrhosis, but there has not been a complete review of all deficiencies so far. 

Deficiency of fat-soluble vitamins is especially frequent in this group of patients. Vitamin D deficiency happens in cirrhosis irrespective of the etiology and is not limited to patients with cholestatic disease. The prevalence of vitamin D deficiency in patients with liver cirrhosis is estimated at 64–92% and increases according to the severity of liver insufficiency, as measured with the Child-Pugh classification [6,7]. Some studies have shown that vitamin D deficiency is associated with liver decompensation, high incidence of infection and increased mortality [8,9,10]. Vitamin A deficiency has been related to immune system disorders and to a more severe liver disease course [5,11]. Vitamin E deficiency has been associated with reperfusion injury after liver transplantation and neuropathies, and vitamin K deficiency, with coagulation disorders [12]. Patients with liver insufficiency have low levels of serum transport proteins, which could potentially lead to overestimation of such deficiencies; however, studies in patients with liver disease in pre-cirrhotic phases suggest that these are real deficiencies.

Patients with liver cirrhosis are also predisposed to water-soluble vitamin deficiencies, especially vitamin B1, irrespective of cirrhosis etiology. Deficiencies of pyridoxine (B6), folate (B9) and cobalamin (B12) are also common, mainly due to decreased liver reserves [13]. However, there are currently few high-quality studies on the true prevalence of these deficiencies and the need for supplementation.

As for trace elements, it is common to find deficiencies of calcium, magnesium, zinc and iron in the serum of cirrhotic patients. Zinc deficit is common in chronic liver diseases, such as chronic hepatitis, metabolic-associated fatty liver disease (MAFLD) and liver cirrhosis. In such patients, zinc deficiency origins many types of metabolic abnormalities, including insulin resistance, hepatic steatosis, iron overload and hepatic encephalopathy. However, these metabolic anomalies may be improved with zinc supplements [14,15]. 

The guidelines from the European Association for the Study of the Liver on nutrition in patients with chronic liver disease published in 2018 recognize that there are no specific studies or evidence on the benefit of micronutrient supplementation in patients with cirrhosis [16]. However, they suggest that confirmed deficiencies should be supplemented in accordance with the general recommendations for usual clinical practice.

Most of the studies evaluating the prevalence of micronutrient deficiencies in decompensated cirrhosis have a small number of subjects, only assess the deficiency of certain micronutrients and are performed on individuals awaiting liver transplant.

The main goal of the present study was to determine the prevalence of micronutrient deficiencies in patients with decompensated liver cirrhosis of any etiology and any degree of liver failure and to assess whether any of these deficiencies correlated with liver disease severity.

## 2. Materials and Methods 

### 2.1. Study Design

This was a prospective single-center cohort study conducted at Parc Taulí Hospital from October 2017 to December 2019.

### 2.2. Sampling and Inclusion Criteria

We included 125 consecutive patients with liver cirrhosis of any etiology admitted to our hospital due to decompensation (ascitic decompensation, infections, hepatic encephalopathy, alcoholic hepatitis, acute kidney injury and portal hypertensive bleeding). We excluded from the analysis all patients who were taking supplements of any of the micronutrients of interest.

### 2.3. Sample Size Calculation

As there were no existing studies on calculating the prevalence of micronutrient deficiencies, it was not possible to estimate a sample size. Therefore, the sample was limited by the period of the study (from October 2017 until December 2019) and the number of admissions to the hepatology unit due to decompensated cirrhosis: 125 patients were included. This sample size allowed us to estimate characteristics related to prevalence of micronutrient deficiencies with a precision of ±10% and 95% confidence, assuming a prevalence of 80% of the variable of interest. 

### 2.4. Data Collection and Measurements

Patients were identified with a number code to ensure confidentiality of their personal data. The following variables were obtained at the time of inclusion: demographic data (age and sex), smoking and alcohol habits, presence of diabetes, anthropometric parameters (weight, height, tricipital skinfold thickness and mid-arm circumference), reason for admission, blood test results (hemogram, bilirubin, international normalized ratio, renal function, albumin, prealbumin, total protein, cholesterol [high-density lipoprotein (HDL) and low-density lipoprotein (LDL)], triglycerides, fat-soluble vitamins (A, D, E, K), water-soluble vitamins (B1, B6, B9 or folic acid, B12 and C) and trace elements (calcium, phosphorus, zinc, magnesium, copper, iron and ferritin)) and cirrhosis etiology (alcoholic, hepatitis C virus (HCV), hepatitis B virus (HBV) or MAFLD cirrhosis). Patients with primary biliary cirrhosis were not included given the low number of decompensations and the bias in the interpretation of deficiency of fat-soluble vitamins. The vitamin forms we measured were the following: retinol (vitamin A), thiamine diphosphate (Vitamin B1), pyridoxal phosphate (vitamin B6), cyanocobalamin (vitamin B12), folic acid (vitamin B9), ascorbic acid (vitamin C), 25-hydroxicholecalciferol (vitamin D), alpha-tocopherol (vitamin E) and phylloquinone (vitamin K).

We also recorded the presence of hepatic complications (ascitic decompensation, infections, hepatic encephalopathy, alcoholic hepatitis, acute kidney injury and portal hypertensive bleeding). We only included patients with alcoholic hepatitis who required admission because they met at least one of the following hospital admission criteria for the disease: hepatic encephalopathy, bilirubin levels >12 mg/dL, prothrombin time >1.36, acute kidney injury, ascites or important social problems. We defined acute kidney injury (AKI) according to the position paper of International Club of Ascites: an increase in serum creatinine ≥0.3 mg/dL within 48 h or a percentage increase in serum creatinine ≥50% from baseline, which have occurred within the prior seven days [17].

From physical examination we obtained patient weight, height, body mass index (BMI), mid-arm circumference, triceps skinfold and mid-arm muscle circumference (MAMC). The severity of liver dysfunction was assessed using the Child-Pugh classification system and the MELD score, in which a higher score indicates more severe liver dysfunction.

### 2.5. Definition of Deficiencies

We defined micronutrient deficiencies according to the hospital laboratory reference values. We considered a deficiency to be any value below the lower limit of normality, except for vitamin D. For vitamin D, we considered a true deficiency to be values below 20 ng/mL, the lower limit of normality being 30 ng/mL. Values between 20 and 30 ng/mL were considered vitamin D insufficiency and values below 10 ng/mL were considered severe deficiency. 

The lower limit of the reference range used for each nutrient were the following: vitamin A (0.3 mg/L) and vitamin E (5 µg/mL), both measured by high-performance liquid chromatography-ultraviolet (HPLC-UV) (Waters®), vitamin B1 (2 µg/dL) and vitamin C (0.4 mg/dL), both measured by high performance liquid chromatography-fluorescence detection (HPLC-FLD) (Waters®), vitamin K (0.13 µg/L), measured by ultra-high performance liquid chromatography tandem mass spectrometry (UHPLC-MS/MS) (Waters®), vitamin B6 (23 nmol/L), measured by enzyme immunoassays (EIA), vitamin D (20 ng/mL), vitamin B12 (150 pg/mL) and folic acid (2 ng/mL), measured by electrochemiluminescence immunoassay (ECLIA) (Roche Diagnostics GmbH®), iron (60 µg/dL) and magnesium (1.6 mg/dL), measured by colorimetric assays (Roche Diagnostics GmbH®), calcium (8.8 mg/dL) and phosphorous (2.7 mg/dL), measured by complexometric assays (Roche Diagnostics GmbH®), ferritin (30 ng/mL), measured by immunoturbidimetric assays (Roche Diagnostics GmbH®), and zinc (68 µg/dL) and copper (70 µg/dL), measured by colorimetric assays (Randox®).

### 2.6. Statistical Methods

Descriptive analysis was performed. Continuous variables were described using mean and standard deviation (SD) or median and interquartile range, as appropriate. For categorical variables, absolute numbers and percentages were calculated with their 95% confidence intervals. We used the T-test for continuous variables and the Chi square test for categorical ones. We collected and analyzed data using SPPS. In all cases, statistical significance was achieved when the *p*-value was smaller than 0.05.

### 2.7. Ethics

The design, procedures and goal of the study were approved by the corresponding ethics review board (Clinical Research Ethical Committee of Parc Taulí Hospital Ref.: 2017536), ClinicalTrial gov identifier: NCT03236038. Participants were given information about the content of the study and signed a consent form before their inclusion. The study complied with the ethics set out in the Declaration of Helsinki. Confidentiality was preserved in agreement with existing Spanish Data Protection Law (15/1999). 

## 3. Results

### 3.1. Patient Characteristics

A total of 125 patients with decompensated liver cirrhosis of various etiologies were included. Baseline patient characteristics are shown in Table 1. The mean age was 62.6 ± 10.3 years, and 76.8% were men. The etiology of liver disease was alcoholic in 79.2%, 57.4% of whom still consumed alcohol. The vast majority of patients recruited had severe liver dysfunction: 12 patients were in Child-Pugh class A (9.6%), 70 were in Child-Pugh class B (56%) and 43 were in Child-Pugh class C (34.4%). The mean MELD score was 16.12 ± 6.3. 

Patients were classified as underweight (1.7%), normal weight (27.1%), overweight (39.8%), obese (24.6%) and extreme obese (6.8%) based on body mass index (BMI). According to mid-arm muscle circumference (MAMC), 8 patients (6.4%) presented malnutrition (defined by MAMC < percentile 10) and 22 patients (17.6%) presented severe malnutrition (defined by MAMC < percentile 5). 

At the time of admission, 80% had ascites, 37.6% had an infection, 24.8% had hepatic encephalopathy, 16.8% had acute kidney injury, 15.2% had portal hypertensive bleeding and 14.4% had alcoholic hepatitis. Of the patients who had infections at the time of admission, only 6.4% had spontaneous bacterial peritonitis (SBP). Respiratory and urinary infections were the most common at 8.8% each. 

### 3.2. Micronutrient Deficiencies

Blood test results including serum levels of trace elements and vitamins are listed in Table 2. 

We detected a high prevalence of deficiency of vitamin A (93.5%), vitamin D (94.5%) and zinc (85.6%). These deficiencies were practically universal in this group of patients. Excluding patients taking vitamin D supplements from the entire cohort, only 1 patient (0.91%) had optimal vitamin D serum levels >30 ng/mL, whereas 82 patients (75.2%) had severe vitamin D deficiency (serum concentration <10 ng/mL). The prevalence of vitamin B6 and vitamin C deficiency was 60.8% and 50.5%, respectively. The prevalence of iron and phosphorous deficiency was also not insignificant: 38.4% and 34.4%, respectively. Copper and magnesium deficiencies were detected in 16.8% and 12.8% respectively, and vitamin E deficit was detected in 15.4%. Plasma concentrations of vitamin E are influenced by lipoprotein metabolism. Due to the low concentrations of cholesterol and triglycerides in some patients, the vitamin E/cholesterol + triglycerides ratio was calculated, which better reflects the nutritional status of this vitamin. The prevalence of calcium and folic acid deficiencies were 4.6% and 5.2% respectively, and for vitamin B1 and vitamin K, were 3.7% and 3.1%. Of the entire cohort, no patient had vitamin B12 deficiency. The prevalence of all deficiencies is summarized in Table 3. It shows the total sample from which the prevalence of each micronutrient deficiency was calculated, as we excluded those who were taking that particular supplement.

Some of the cut-offs we used to establish some vitamin deficiencies were different from the literature. Vitamin A could have been over-reported since we used a cut-off for deficiency of 0.3 mg/L (1.07 µmol/L), whereas other authors used 0.196 mg/L (0.7µmol/L), values below which we considered as a severe deficit. However, this has little impact on the prevalence as only 4 patients had vitamin A levels between 0.196 and 0.3mg/L, so the resulting prevalence would be 90.2%. The same occurred for vitamin C. We used a cut-off for deficiency of 0.4 mg/dL instead of 0.2 mg/dL. In this way, since there were 6 patients who had vitamin C levels between 0.2 and 0.4 mg/dL, the resulting prevalence would be 44.6%. On the contrary, vitamin K deficit could have been under-estimated since we used a cut-off for deficiency of 13 µg/L instead of 15 µg/L, as described in the literature. In this case, two patients had vitamin K levels of 0.14 µg/L, so the resulting prevalence would be 5.1%.

**Figure 1:**
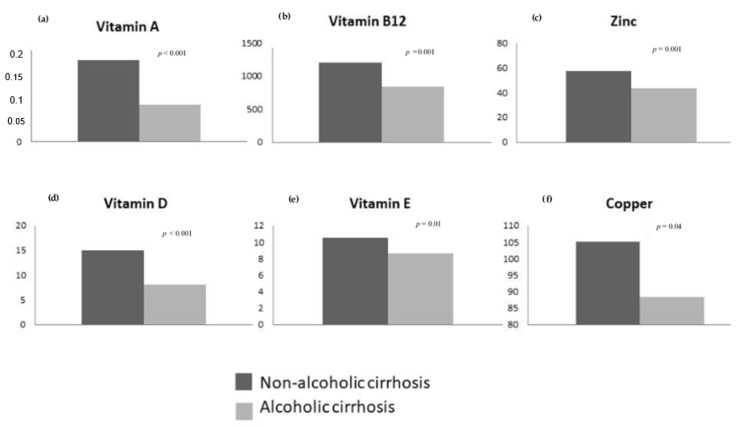
Bar chart of the serum levels of the micronutrients that reached statistical significance when analyzed depending on etiology of cirrhosis as a dichotomic variable. (**a**) Etiology of cirrhosis and vitamin A levels. (**b**) Etiology of cirrhosis and vitamin B12 levels. (**c**) Etiology of cirrhosis and zinc levels. (**d**) Etiology of cirrhosis and vitamin D levels. (**e**) Etiology of cirrhosis and vitamin E levels. (**f**) Etiology of cirrhosis and copper levels.

There were no significant differences in micronutrient deficiencies according to age, sex, type of cirrhosis decompensation, current alcohol consumption, presence of diabetes mellitus or body mass index. Likewise, there were no significant differences in relation to the etiology of cirrhosis. However, since most of our patients had alcoholic cirrhosis, we analyzed the micronutrient levels depending on the etiology of cirrhosis by treating it as a dichotomous variable: alcoholic cirrhosis (*n* = 107) vs. non-alcoholic cirrhosis (*n* = 18). Then, we found statistically significant differences for serum levels of vitamin A (0.1 ± 0.09 vs. 0.23 ± 0.1 (*p* < 0.001)), vitamin B12 (861.5 ± 455 vs. 1160.5 ± 594.8 (*p* = 0.001)), vitamin E (8.3 ± 4.3 vs. 10.7 ± 2.7 (*p* = 0.01)), vitamin D (8.1 ± 4.6 vs. 14.9 ± 11.4 (*p* < 0.001)), copper (88.8 ± 32.2 vs. 105.06 ± 29.5 (*p* = 0.04)) and zinc (44.06 ± 33.1 vs. 58.6 ± 17.8 (*p* = 0.001)). These results are shown in Figure 1.

With regard to liver function, we identified that patients in Child-Pugh class C had lower values of vitamin A (*p* < 0.0001), zinc (*p* < 0.0001), vitamin E (*p* = 0.01) and magnesium (*p* = 0.05). In addition, Child-Pugh class C patients had higher levels of iron (*p* = 0.009), ferritin (*p* = 0.002) and vitamin B12 (*p* < 0.0001) than those in class A and B. The median serum concentration of vitamin A in patients in Child-Pugh class C was 0.05 mg/L, whereas the median values for class A and B were 0.24 and 0.09 mg/L, respectively. As for zinc, we found that patients in Child-Pugh class C had a median serum value of 35 µg/dL, while those in class A and B had a median serum concentration of 56 and 45.5 µg/dL. These results are summarized in Table 4.

**Figure 2:**
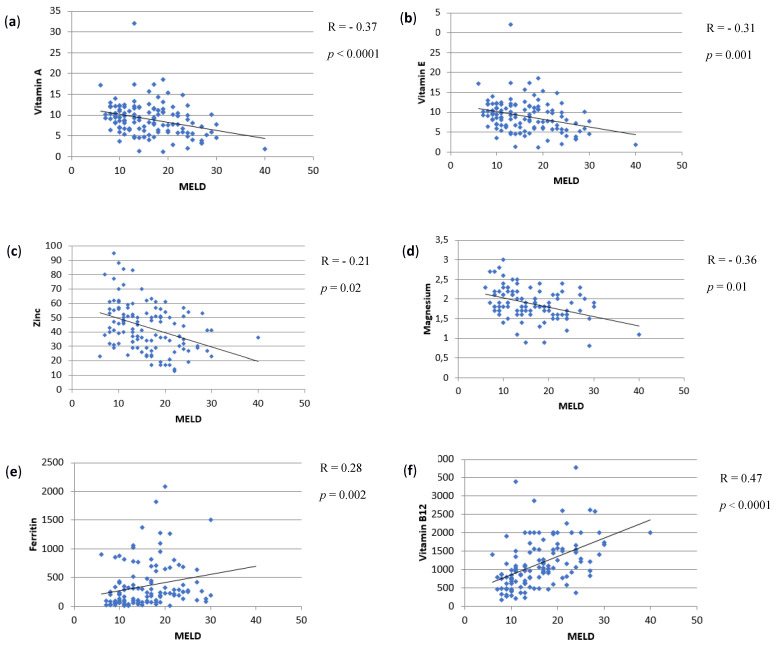
Scatter plots of the correlations of MELD score with the micronutrients that reached statistical significance. R is the correlation coefficient. (**a**) MELD score and vitamin A (negative correlation, R = –0.37), (**b**) MELD score and vitamin E (negative correlation, R = –0.31), (**c**) MELD score and zinc (negative correlation, R = –0.21), (**d**) MELD score and magnesium (negative correlation, R = –0.36), (**e**) MELD score and ferritin (positive correlation, R = 0.28), (**f**) MELD score and vitamin B12 (positive correlation, R = 0.47).

Patients with a higher MELD score had lower levels of vitamin A (*p* < 0.0001), vitamin E (*p* = 0.001), magnesium (*p* = 0.01) and zinc (*p* = 0.02), and higher values of ferritin (*p* = 0.002) and vitamin B12 (*p* < 0.0001). These results are represented in Figure 2.

As for infections, we analyzed whether any micronutrient deficiencies correlated with infection at the time of admission. We did not find a statistically significant correlation between any vitamin deficiency and the presence of infection. However, when we focused on trace elements, we found that patients who had infection at the time of admission had lower serum concentrations of phosphorus, and this correlation reached statistical significance with a *p*-value of 0.03.

We also analyzed if patients with active alcohol consumption had a different micronutrient deficiency profile from those without active alcohol consumption. We found that patients with active consumption had lower serum concentrations of vitamin A (*p* = 0.001), vitamin E (*p* = 0.02) and vitamin D (*p* < 0.0001), and higher serum concentrations of vitamin B12 (*p* = 0.013).

## 4. Discussion

Vitamins and minerals play a significant role in several antioxidant, anti-inflammatory and anti-apoptotic metabolic processes, and their deficiencies are common in patients with cirrhosis regardless of the etiology of the liver disease. Our study is the first to provide an overall view of the prevalence of micronutrient deficiency for both vitamins and trace elements in patients with decompensated cirrhosis. So far, this is the largest and most complete prevalence study of micronutrient deficiency in patients with decompensated cirrhosis, regardless of their status on the transplant list. To date, the most studied and well-documented deficiencies in patients with liver disease are those of zinc and vitamin D. 

Zinc deficiency may occur as a consequence of diuretic use or restricted animal protein intake [18,19,20]. The results of our study showed a high prevalence—around 85%—of zinc deficiency, which is similar to previously published findings by several authors (83–94%) [21,22,23]. In 1996, Pescovitz et al. conducted a study in 34 cirrhotic patients awaiting liver transplantation and found a prevalence of zinc deficiency of 94% [22]. More recently, in 2015, Sengupta et al. found a high prevalence of zinc deficiency (83%) in 163 cirrhotic patients [23]. They also demonstrated that the deficiency was significantly higher in patients with Child-Pugh class B or C cirrhosis than in those in Child-Pugh class A. These results are consistent with ours: we found a high prevalence of zinc deficiency (85.6%) and a significantly higher deficiency in patients with more severe liver disease, with a median zinc concentration of 56, 45.5 and 35 µg/dL in patients in Child-Pugh class A, B and C, respectively. Patients in Child-Pugh class C had significantly lower values of zinc than those in class A and B (*p* < 0.0001). The results were similar when we considered the MELD score: we found a correlation between patients with higher MELD score and lower values of zinc (*p* = 0.02).

Some authors have demonstrated that vitamin D deficiency has significant implications in chronic liver disease: it correlates with disease severity and degree of fibrosis and is associated with poorer outcomes [11,24,25,26,27]. A study in 88 hospitalized cirrhotic patients conducted by Anty et al. found a prevalence of severe vitamin D deficiency (<10 ng/mL) of 56.8% [28]. They also described that infections were more frequent in this group than in those with higher levels of vitamin D (54% vs. 29%, *p* = 0.02). They concluded that severe vitamin D deficiency was a predictor of infection independently of Child-Pugh class. 

Similarly, Buonomo et al. found a prevalence of vitamin D deficiency of 64% in a group of 345 inpatients and outpatients, 50.27% of whom had a severe deficiency [29]. They also described an association between severe vitamin D deficit and poor survival, independently of Child-Pugh class.

These results fit in with ours: we found a prevalence of vitamin D deficiency of 94.5% and a prevalence of vitamin D insufficiency (<30 ng/mL) of 99%. Eighty-two patients (75.2%) had a severe deficiency (<10 ng/mL). However, we did not find a correlation between vitamin D deficiency and more advanced liver disease as measured with the MELD score or Child-Pugh classification.

In 2013, Venu et al. conducted a retrospective study in 63 outpatients awaiting liver transplant and reported a high prevalence of vitamin A and D deficiency (69.8% and 80.9%, respectively) [5]. However, only 3.2% of the patients had a vitamin E deficiency.

In the present study in hospitalized patients, we found a prevalence of vitamin A deficiency of 93.5% and a prevalence of vitamin E deficiency of 15.4%. Vitamin K deficiency was observed in 3.1% of patients. These results suggest that prolonged prothrombin time in liver diseases is not due to vitamin K deficiency but rather to liver insufficiency, since the vast majority of the patients in the study had a prolonged prothrombin time but only a few had an objective vitamin K deficiency. Vitamin K deficiency was not greater in the subgroups with active alcohol consumption or lower prealbumin serum levels.

Interestingly, the patients with the most severely impaired liver function as measured with MELD score had significantly lower levels of vitamin A (*p* < 0.0001) and vitamin E (*p* = 0.001). In addition, we found that patients with marked advanced liver disease (Child-Pugh C) also had lower values of vitamin A (*p* < 0.0001) and E (*p* = 0.01) than those with more preserved liver function (Child-Pugh A and B). Low vitamin A levels can be attributed to reduced synthesis of prealbumin and retinol-binding protein in the liver. Furthermore, alcoholic cirrhosis is usually associated with zinc deficit, which can contribute to decreased retinol-binding protein synthesis and, therefore, impair vitamin A mobilization [30].

Another point to be interpreted with caution is that of low vitamin E levels, because these are influenced by lipoprotein metabolism. Due to the low serum concentrations of cholesterol and triglyceride in these patients, the vitamin E/cholesterol + triglycerides ratio should be calculated, since it better reflects the nutritional status of this vitamin. 

For water-soluble vitamins, we found a prevalence of vitamin B6 and vitamin C deficiency of 60.8% and 50.5%, respectively. Vitamin B1 deficiency was observed in 3.7%, and none of the patients had B12 vitamin deficiency. In fact, 74% of the patients had serum levels of vitamin B12 above the upper limit of normality with a median value of 982 pg/mL and a range of 664–1542. Strikingly, we found that patients in Child-Pugh class C had higher values of vitamin B12 than those in class A or B (*p* < 0.0001). The results were similar when using MELD score: patients with higher MELD score had higher serum values of vitamin B12 (*p* > 0.0001).

Our results are consistent with those reported by Sugihara et al. [31], who found that patients in Child-Pugh class C had serum vitamin B12 levels significantly higher than those in class A or B. Specifically, they found a serum concentration of 1308 ± 599 pg/mL in Child-Pugh class C patients, 660 ± 464 pg/mL in class B and 784 ± 559 pg/mL in class A (*p* = 0.029). Furthermore, they found that higher values of vitamin B12 were linked to poor prognosis. 

One finding from our study that may be impactful is that the prevalence of vitamin B12 deficiency was 0% in a population of patients with cirrhosis mostly of alcoholic etiology. The association of vitamin B12 deficiency with chronic alcoholism has been known for over 40 years [32,33]. However, more recent studies comparing patients with liver disease and healthy subjects both with excessive chronic consumption of alcohol found that the presence of liver damage correlated with elevated vitamin B12 levels. Therefore, they concluded that levels of vitamin B12 were a good indicator of the degree of hepatocytes injury by alcohol. They proposed two mechanisms that may explain this fact: firstly, excess release due to the destruction of liver cells, and secondly, reduced uptake of vitamin B12 from serum by injured hepatocytes [31,34,35].

In relation to trace elements, the prevalence of iron and phosphorous deficiency was also high: 38.4% and 34.4%, respectively. Interestingly, however, we found that Child-Pugh class C patients had higher serum iron (*p* = 0.009) and ferritin (*p* = 0.002) values than those in class A and B. Similar results were found when we analyzed based on the MELD score: patients with the most severe liver dysfunction had higher values of ferritin (*p* = 0.002). In fact, these results are consistent with several studies conducted in patients with decompensated liver disease, which showed an association between serum ferritin levels and patient prognosis [36,37,38,39]. Walker et al. assessed 191 candidates for liver transplant and reported that baseline serum ferritin levels >200 µg/L was an independent predictor of mortality at 6 and 12 months [38]. Similarly, Maiwall et al. carried out a study with 318 patients with decompensated cirrhosis and found that ferritin values correlated with severity of liver decompensation and with early liver-related death, independent of the MELD score [39]. They described a progressive risk of death with increasing ferritin levels which plateaued and rose very gradually above a concentration of 500 µg/L. In our study, the median serum concentrations and the range were 71 (12–774), 208.5 (10–2083) and 260 (12–4262) µg/L for Child-Pugh class A, B and C, respectively.

As for copper and magnesium, deficiencies were detected in 16.8% and 12.8%, respectively. In our bibliographic search, we did not find any studies on the prevalence of copper or magnesium deficiency in patients with cirrhosis or liver disease. 

Some authors have reported the benefits of screening for magnesium and copper deficiency and of supplementation if a deficiency is detected. A review by Liu et al. concluded that magnesium deficiency aggravates cirrhosis and could contribute to liver cancer progression [40]. They stated that magnesium supplementation can slow the progression of liver disease and decrease the associated mortality. Nangliya et al. carried out a study with 150 cirrhotic patients and 50 controls and reported that serum magnesium, zinc and selenium levels were significantly decreased in cirrhotic patients in comparison to controls (*p* < 0.001) [41]. In our study, we found that patients with more advanced liver disease as measured with MELD score had lower serum concentrations of magnesium (*p* = 0.01). Similarly, Child-Pugh class C patients had lower serum concentrations of magnesium than Child-Pugh class A and B patients (*p* = 0.05).

As for copper, Aigner et al. conducted a study with 120 patients with MAFLD and concluded that an association between copper status and iron homeostasis exists, suggesting that low copper bioavailability may increase hepatic iron stores [42], which could contribute to the progression of liver fibrosis [43,44]. 

In our study, the prevalence of calcium and folic acid deficiency was 4.6% and 5.2%, respectively. It is well-known that patients with liver disease develop bone loss that can lead to atraumatic fractures. Nakchbandi recommended that physicians should evaluate bone density in these patients regardless of serum calcium values and should supplement with a minimum of vitamin D and calcium to prevent bone fractures and osteoporosis [45]. 

As the prevalence of micronutrient deficiencies in cirrhotic patients is so high, some authors recommend checking serum magnesium, zinc, vitamin D and vitamin A every 6 months [11,20]. We fully agree with them and support the need for micronutrient analyses in these patients to allow supplementation to be given as soon as possible. Given the high prevalence of vitamin A, vitamin D and zinc deficiencies detected and the safety of their supplementation, it could be reasonable to start treatment at the time of admission at the established dosage and continue with further supplementation during hospitalization if other deficiencies are detected. However, the efficacy of this intervention should be evaluated in subsequent studies.

Finally, an issue that should be discussed is that in the present study, micronutrient levels were not corrected for inflammatory status. Inflammation can confuse the interpretation of micronutrient status, and one of the key elements involved in cirrhosis pathophysiology is systemic inflammation [46]. However, this chronic inflammation is difficult to assess and quantify. In fact, most studies on inflammatory status and micronutrients conducted so far only focus on acute inflammatory response. In 2017, researchers from the Biomarkers Reflecting Inflammation and Nutritional Determinants of Anemia (BRINDA) project published a new mathematical method to estimate the prevalence of iron and vitamin A in settings of acute and chronic inflammation [47]. They suggested that without this adjustment, iron deficiency could be under-reported and vitamin A and zinc deficiency could be over-reported [47,48,49]. Nevertheless, it is unclear if the results of this study can be used in individuals. There is still a lot of research to be done in this area, especially to assess the benefits of micronutrient supplementation in patients with chronic systemic inflammation, such as those with liver cirrhosis.

## 5. Conclusions

This study provided a detailed picture of the micronutrient deficiency situation in patients with decompensated cirrhosis mostly of alcoholic etiology who we see every day in our hospitals, regardless of their age and the severity of liver failure. The most common vitamin deficiencies were A, D, B6 and C. Zinc deficiency was practically universal in this population. The severity of liver disease correlates well with lower levels of magnesium, zinc, vitamin A and vitamin E, and with higher serum values of vitamin B12 and ferritin. Further studies assessing the global supplementation of all these microelements in patients with cirrhosis and their effects on prognosis should be explored in the near future.

## Figures and Tables

**Table 1:** Summary of baseline characteristics for all included patients.

Variable	Value
Age	62.6 ± 10.3
Sex Male Female	96 (76.8%) 29 (23.2%)
Current smoker	52 (41.6%)
Current alcohol consumption	70 (57.4%)
Diabetes mellitus	45 (36%)
Child-Pugh class A B C	12 (9.6%) 70 (56%) 43 (34.4%)
MELD score	16.2 ± 6.3
Mid-arm muscle circumference <percentile 5 <percentile 10 ≥percentile 10 BMI categories Underweight (<18.5) Normal weight (18.5–24.9) Overweight (25–26.9) Obese (30–39.9) Extremely obese (>40)	22 (17.6%) 8 (6.4%) 95 (76%) 2 (1.7%) 32 (27.1%) 47 (39.8%) 29 (24.6%) 8 (6.8%)
Etiology of cirrhosis Alcohol Hepatitis C Alcohol + hepatitis C Hepatitis B MAFLD Cryptogenic	99 (79.2%) 5 (4%) 8 (6.4%) 2 (1.6%) 4 (3.2%) 7 (5.6%)
Decompensation of cirrhosis Ascites Encephalopathy Portal hypertensive bleeding Acute kidney injury Alcoholic hepatitis Infection SBP Respiratory infection Urinary infection Cutaneous infection Bacteremia Other	100 (80%) 31 (24.8%) 19 (15.2%) 21 (16.8%) 18 (14.4%) 47 (37.6%) 8 (6.4%) 11 (8.8%) 11 (8.8%) 7 (5.2%) 5 (4%) 5 (4%)

Data are mean ± standard deviation (SD) for quantitative variables and *n* (%) for qualitative variables. MELD: Model for end-stage liver disease; BMI: Body mass index; MAFLD: Metabolic associated fatty liver disease; SBP: Spontaneous bacterial peritonitis.

**Table 2:** Baseline levels for all patients.

Parameter	Value	Reference Value
Leucocytes	6.86 (1.76–27.06)	4–11 × 10 ^9^/L
Hemoglobin	105.7 ± 26.7	130–175 g/L
Platelets	110 (21–466)	130–400 × 10 ^9^/L
INR	1.47 ± 0.3	0.8–1.3
Urea	45.19 ± 31.7	10–50 mg/dL
Creatinine	1.05 ± 0.56	0.70–1.20 mg/dL
Sodium	136.8 ± 8.4	136–145 mEq/L
Bilirubin	3.4 ± 1.7	0.1–1.3 mg/dL
Total protein	62.07 ± 10.7	66–87 g/L
Albumin	30.1 ± 5.7	34–48 g/L
Prealbumin	8.5 ± 4.2	20–40 mg/dL
Total cholesterol HDL LDL	114.3 ± 49.3 34.3 ± 18.2 64.1 ± 40.6	150–200 mg/dL 35–100 mg/dL
Triglycerides	87.2 ± 47.2	50–200 mg/dL
Vitamin A	0.09 (0.05–0.16)	0.3–1 mg/L
Vitamin B1	3.9 (2.9–13.8)	2–7.2 µg/dL
Vitamin B6	19 (9.05–33.5)	23–173 nmol/L
Vitamin B12	982 (664–1542)	150–695 pg/mL
Vitamin C	0.4 (0.11–0.81)	0.4–2 mg/dL
Vitamin D	6.8 (5.2–49)	>30 ng/mL
Vitamin E	8.8 (5.9–11.1)	5–20 µg/mL
Vitamin K	0.7 (0.2–3.1)	0.13–1.50 µL/L
Folic acid	5.4 (4–9.4)	2–14.54 ng/mL
Corrected calcium for albumin	9.3 ± 0.56	8.8–10.2 mg/dL
Phosphorus	2.88 ± 0.82	2.7–4.5 mg/dL
Magnesium	1.9 ± 0.41	1.6–2.6 mg/dL
Copper	91.3 ± 32.1	70–140 µg/dL
Zinc	45.9 ± 29.1	68–120 µg/dL
Iron	81.4 ± 63.7	60–158 µg/dL
Ferritin	211 (75.5–4362.7)	30–400 ng/mL

Data are mean ± SD or median (range). Albumin (*n* = 87), vitamin B1 (*n* = 81), vitamin D (*n* = 109), vitamin K (*n* = 97), corrected calcium for albumin (*n* = 115). INR: international normalized ratio; HDL: high-density lipoprotein; LDL: low-density lipoprotein.

**Table 3:** Prevalence of micronutrient deficiencies.

Micronutrient (n)	Prevalence
Vitamins A (n = 123) B1 (n = 81) B6 (n = 120) B9 (folic acid) (n = 97) B12 (n = 123) C (n = 101) D (n = 109) E (n = 123) K (n = 97)	115 (93.5%) 3 (3.7%) 73 (60.8%) 5 (5.2%) 0 (0%) 51 (50.5%) 103 (94.5%) 19 (15.4%) 3 (3.1%)
Trace elements Calcium (n = 87) Phosphorus (n = 125) Magnesium (n = 125) Copper (n = 125) Iron (n = 125) Ferritin (n = 125) Zinc (n = 125)	4 (4.6%) 43 (34.4%) 16 (12.8%) 21 (16.8%) 48 (38.4%) 6 (5.2%) 107 (85.6%)

**Table 4:** Serum concentrations of micronutrients according to Child-Pugh class. Univariate analysis.

	Child-Pugh A	Child-Pugh B	Child-Pugh C	*p*-value
Vitamin A	0.24 (0.09–0.59)	0.09 (0.01–0.55)	0.05 (0.02–0.22)	<0.0001
Vitamin B1	4.2 (3.4–4.8)	3.9 (1.2–8.7)	3.1 (1.2–13.8)	0.5
Vitamin B6	27.4 (11.3–106)	22.1 (9–97.8)	12.35 (9–46)	0.21
Folic acid (B9)	6.2 (4–14.40)	5.4 (1.3–20)	5.3 (1.5–23.30)	0.5
Vitamin B12	416.5 (164–1919)	872.5 (269–2868)	1459 (186–3771)	<0.0001
Vitamin C	0.6 (0.1–1.41)	0.41 (0.1–2.69)	0.24 (0.1–9)	0.82
Vitamin D	9.8 (5.5–22.9)	6.6 (5–26.9)	6.6 (5–49)	0.25
Vitamin E	10.6 (4.7–17.4)	9.4 (1.2–32)	6.5 (1.3–14.3)	0.01
Vitamin K	1.14 (0.18–4.03)	0.83 (0.09–4.31)	0.52 (0.05–1.90)	0.07
Calcium	9.2 (8–9.8)	9,4 (8.29–10.8)	9.5 (8.17–10.6)	0.26
Phosphorus	3.2 (2.3–4)	2.75 (1–4,7)	2.75 (1–3.5)	0.27
Magnesium	2.1 (1.8–2.5)	1.8 (0.9–2.7)	1.75 (0.8–2.5)	0.05
Copper	86 (67–126)	96.5 (17–121)	80.5 (40–155)	0.44
Zinc	56 (51–84)	45.5 (17–95)	35 (13–57)	<0.0001
Ferritin	71 (12–774)	208.5 (10–2083)	260 (12–4262)	0.002
Iron	32 (25–269)	46.5 (11–205)	80 (22–178)	0.009

Data are median (range). *p*-value (Child-Pugh C compared to Child-Pugh A and B).

## Data Availability

The datasets generated and analyzed during the current study are not publicly available due to provisions of the written informed consent.
